# Death of Thomas Hawkes Tanner

**Published:** 1871-09

**Authors:** 


### Death of Thomas Hawkes Tanner.

Thomas Hawkes Tanner, M.D., F.L.S., M.R.C.P., died in Brighton, July 7th, at the early age of 46. He began practice in London in 1847, and since then had held several responsible appointments, and enjoyed for many years past a very extensive practice. His "Manual of the Practice of Medicine,' originally published in 1864, has gained in popularity with each succesive edition, and from a mere pocket manual has grown to a complete work in two large volumes. He was the author also of "Signs and Diseases of Pregnancy," a "Practical Treatise on the Diseases of Infancy ond Childhood," "Clinical Medicine," an "Index of Diseases," "Memoranda on Poisons," and several smaller works the papers on various subjects. The leading characteristic of his life was indomitable industry, which enabled him to accomplish a vast amount of work, though at the sacrifice of his own health. He had been suffering for several years from renal disease consequent on an attack of scarlet fever in 1854, and his death was caused by uræmia. For two months before his death he had been obliged to relinquish his professional duties.—*New York Medical Journal.*

